# Non-Targeted Metabolomic Analysis of the Kombucha Production Process

**DOI:** 10.3390/metabo12020160

**Published:** 2022-02-08

**Authors:** Thierry Tran, Rémy Romanet, Chloé Roullier-Gall, François Verdier, Antoine Martin, Philippe Schmitt-Kopplin, Hervé Alexandre, Cosette Grandvalet, Raphaëlle Tourdot-Maréchal

**Affiliations:** 1 UMR Procédés Alimentaires et Microbiologiques, Institut Agro Dijon, Université de Bourgogne Franche-Comté, 21000 Dijon, France; remy.romanet@u-bourgogne.fr (R.R.); chloe.roullier-gall@u-bourgogne.fr (C.R.-G.); rvalex@u-bourgogne.fr (H.A.); cosette.grandvalet@u-bourgogne.fr (C.G.); tourdot@u-bourgogne.fr (R.T.-M.); 2 Biomère, 14 Rue Audubon, 75120 Paris, France; fverdier@jubiles.bio (F.V.); amartin@jubiles.bio (A.M.); 3 Comprehensive Foodomics Platform, Technische Universität München, 85354 Freising, Germany; schmitt-kopplin@helmholtz-muenchen.de; 4 Research Unit Analytical BioGeoChemistry, Department of Environmental Sciences, Helmholtz Zentrum München, 85764 Neuherberg, Germany

**Keywords:** kombucha, metabolomics, process, fermentation, tea, polyphenols

## Abstract

Kombucha is a traditional fermented beverage obtained from the transformation of sugared black tea by a community of yeasts and bacteria. Kombucha production recently became industrialized, but its quality standards remain poorly defined. Metabolomic analyses were applied using FT-ICR-MS to characterize the impacts of production phases and the type of tea on the non-volatile chemical composition of kombucha. Independently from tea type, the first phase of acidification in open vessel was characterized by the release of gluconate and gallate from acetic acid bacteria metabolism and probably from polymeric polyphenols, respectively. The second phase of carbonation in closed vessel induced a consumption or transformation of oleic acid that could be consecutive of oxygen limitation. The first phase had the most impact on molecular diversity, but tea type mainly influenced the global composition in polyphenol profile. Black tea polyphenols were more impacted by microbial activity compared to green tea polyphenols.

## 1. Introduction

Metabolomics has undergone an important development in the field of food and nutrition sciences [1]. It is defined as the high-throughput identification and quantification of small molecules (metabolites) from different molecular families and constitutive of a metabolome. The study of metabolomes in food aims at determining their composition by analyzing the highest number of compounds possible, which is why this approach is often coupled with non-targeted methods. A deep and comprehensive understanding of food composition allows the identification of parameters, markers, and signatures linked to the identity or authenticity of a given food, thus helping defining it and distinguishing from others, or adulterated versions [2]. Indeed, the identity of food products is closely tied to their production process. In the context of fermented beverages, metabolomics was successfully applied to wine or beer, and allowed to determine the impacts of vintage and terroir on the composition of Burgundy red wines, or highlight the role of starch source on the metabolomes of German beers [3,4].

Therefore, such an approach is of great use when it comes to foods or beverages that are poorly characterized and raises many problematics regarding their production and quality management. This relevantly applies to kombucha, a traditional fermented beverage from sugared tea infusion formerly produced at home and now experiencing high market development as a commercialized product [5]. Its putative health benefits are promoted but conclusive clinical trials remain to be conducted to confirm them [6,7]. Kombucha is produced by inoculating a sugared tea infusion with a microbial consortium composed of yeasts and bacteria, mainly acetic acid bacteria, which can occur as multiple compositions in species [8]. The production process of kombucha occurs mainly as following: during a first phase in open vessel, yeasts with invertase and fermentative activity convert sucrose in glucose and fructose, which are in turn converted in ethanol and carbon dioxide through alcoholic fermentation. Acetic acid bacteria use those yeast metabolites to produce acetic and gluconic acids, thus acidifying the matrix from the consumption of sugars. When satisfying acidity is reached, bottling or tight sealing of the vessel induces oxygen deprivation and retention of carbon dioxide and natural carbonation is thus achieved [9]. In our previous study, the bottling induced further sucrose hydrolysis and no significant increase of total acidity was observed in relationship to restricted oxygen access [9]. While this main metabolic scheme was significantly investigated, many grey areas remain associated with this complex microbiological process, involving interkingdom and interspecies interactions in a very unusual matrix. Metabolomics can help raise new hypotheses and uncover metabolites linked to biological activities or the transformations occurring during the process [10].

Metabolomics has already been used to study kombucha. In the study of Villarreal-Soto et al. (2020) [11], three different kombucha consortia were analyzed by HPLC-DAD and GC/MS in addition to metagenomics. The different microbial compositions led to different microbial activities and chemical compositions. Phenolic compounds such as phenolic acids or catechin were reported to be impacted by microbial activity. Moreover, some concentration in metabolites varied significantly across the consortia, such as propanoic acid, 2-phenylethanol, as well as carbohydrates. The establishment of links between chemical composition and antioxidant, anti-inflammatory and antiproliferative activities was also attempted. A similar approach was employed in the study of Savary et al. (2021) [12], with the coupling of Quadrupole Time of Flight Liquid Chromatography/Mass Spectrometry (LC-Q-TOF/MS), enzymatic kits and GC/MS with metagenomics to follow changes in metabolites throughout a 27-day kombucha production process in open vessel. Microbial dynamics could then be linked to microbial activities in terms of kinetics of production of organic acids and volatile compounds. Several phases could be characterized based on microbial dynamics and specific metabolite compositions using clustering and multiple factor analyses. While these two studies relied on targeted chemical analysis, the study of Cardoso et al. (2020) [13] used LC-Q-TOF/MS as non-targeted analytical method with a focus on phenolic compounds. In this study, black and green tea kombucha phenolic profiles were compared to corresponding unfermented teas. Results showed higher diversity of polyphenols in black tea kombucha than in green tea kombucha, which corresponded also to a higher antioxidant activity in black tea kombucha, highlighting the significant impact of the type of tea used on the kombucha’s phenolic profile. So, the utilization of untargeted techniques is shown to greatly expand the knowledge on the metabolic landscape of kombucha. One of those techniques, Fourier–Transform–Ion Cyclotron Resonance/Mass Spectrometry (FT-ICR/MS) has been successfully used for the analysis of tea metabolites for the unravelling of thearubigins formation [14].

This study aims at analyzing kombucha samples for the first-time with FT-ICR/MS, a non-targeted analytic tool, to determine the impact of production phases (open and closed vessel) from sugared tea to the finished product, as well as the effect of the tea type on the kombucha non-volatile metabolome. Fixed parameters are the kombucha microbial consortium and the preparation of sugared tea infusions (same amount of sugar and tea, whether green or black). The evaluation of the effects focused on the comparison of non-volatile molecular diversity (number of detected features), variations in signal intensity and determination of markers or pathways in association to the conditions studied for their characterization.

## 2. Results and Discussion

### 2.1. Chemical Composition of Samples 

Sugared tea samples possessed a total sugar content of 59.0 ± 1.0 g L^−1^, a pH value of 6.90 ± 0.01 and a total acidity inferior to 1 meq L^−1^. Kombucha samples at D7 possessed a total sugar content of 51.0 ± 10.0 g L^−1^, a pH value of 4.2 ± 0.1 and a total acidity of 20 ± 1 meq L^−1^. Kombucha samples at D12 possessed a total sugar content of 46.4 ± 4.0 g L^−1^, a pH value of 3.84 ± 0.5 and a total acidity between 28 ± 2 and 41 ± 3 meq L^−1^. Important discrepancies in sugar contents, pH and total acidity values of inoculated samples can be explained by biological variability of microbial activities.

### 2.2. Comparison of General Chemical Compositions and Data Visualization 

**Figure 1:**
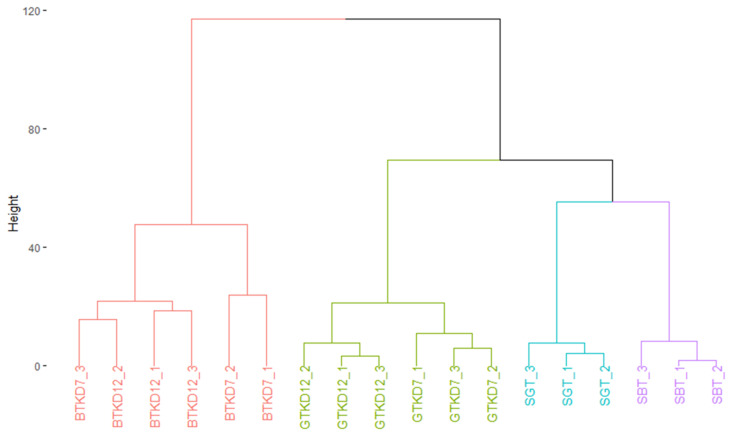
Dendrogram showing composition similarities between the three replicates of sugared black tea (SBT), sugared green tea (SGT) and corresponding kombucha samples (BTK and GTK) at day 7 (D7) and day 12 (D12), structured after Hierarchical Clustering Analysis.

Peak intensities associated to ion masses detected in sugared tea and kombucha samples were analyzed using HCA after data treatment to display similarity in compositions (Figure 1). Clusters separated mainly BTK samples from the others and secondarily GTK samples from sugared teas (SBT and SGT). Microbial activity significantly impacted the chemical composition of the matrix. The type of tea also had an effect, since the composition of GTK samples appeared to be closer to sugared teas and thus less impacted. The production phases showed minor impacts on the chemical composition, with separation of D7 and D12 samples inside BTK and GTK clusters, especially for GTK. P1 et P2 phases were clearly separated for GTK, whereas only one BTKD7 replicate grouped BTKD12 cluster. The next step of our approach attempted to explain the formation of those clusters.

**Figure 2:**
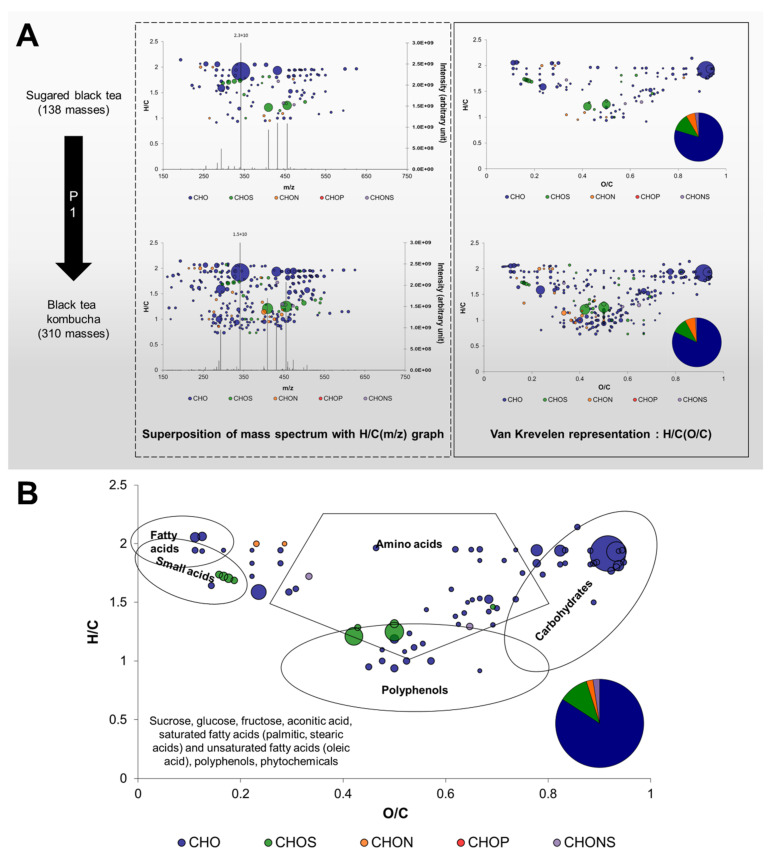
Construction of graphic representations of FT-ICR-MS data. (**A**) From mass spectra, annotation of elemental formula allows to arrange each formula according to elemental ratios (for example H/C) and to distinguish different elemental composition using colors. By arranging formulae using H/C and O/C ratios, Van Krevelen diagrams are obtained and can be complemented with the proportions in different elemental compositions. Enrichment in composition between Sugared Black Tea and corresponding kombucha during the first phase of production (P1) can be observed. (**B**) Van Krevelen diagram of common formula across Sugared Teas and kombuchas at day 7 and day 12, complemented with the proportions in different elemental compositions. The utilization of Van Krevelen diagrams allows the determination of regions corresponding to chemical families such as lipids, small acids, amino acids, polyphenols or carbohydrates, based on the analysis of a large number of compounds [15]. The surface of bubbles expresses relative formulae intensity. With a comparison of mass lists with databased such as METLIN, putative compound identities can be obtained. Most probable candidates were added in the bottom left-hand corner of the diagram.

To achieve that, it is necessary first to define terms and the way data are represented. The approach is detailed in Figure 2. 

Direct infusion FT-ICR-MS was used for metabolite profiling, enabling a wide dynamic range in intensity (10^6^) and we focused on the most abundant compounds (S/N > 4). A peak obtained on mass spectra after FT-ICR-MS data processing associates an ion mass (*m*/*z*) to an intensity, thus providing semi-quantitative data (Figure 2A). For example, more masses were detected in BTK at D7 (310) compared to SBT (138), which corresponded to an enrichment in molecular diversity induced by the inoculation and microbiological activity of P1. Based on highly precise measured m/z values, each value was annotated with a molecular or elemental formula, which distinguishes FT-ICR-MS from other tools for metabolomics. The formulae obtained in the CHONS-space can then be visually represented with different colors based on their elemental compositions and spatially using elemental ratios, for example H/C. Moreover, relative intensities can be expressed through the surface of the bubbles associated to each formula. The utilization of H/C and O/C ratios corresponds to the Van Krevelen diagram, and it can be complemented with a diagram of the proportion in elemental compositions. Figure 2B represents the 99 formulae common to all samples and this group was labeled as “core metabolites” in a Van Krevelen diagram. This representation gives more information on the formulae, since it allowed the determination of zones corresponding to chemical families such as lipids, amino acids, carbohydrates and polyphenols [15]. The core metabolites group included mainly CHO formulae and CHOS, CHON and CHONS in lesser proportion. In addition, different chemical families were represented, such as lipids, amino acids, carbohydrates, and polyphenols. By comparing the masses with databases such as METLIN used in the present study, it was possible to annotate them with putative identities (level 3 annotation level; Table S2) [16,17]. In the case of the core metabolites group, putative identities from the sugared tea matrix could be obtained, such as sucrose, glucose, fructose, palmitic, stearic, and oleic acids along with various polyphenols and phytochemicals (plant compounds).

Additionally, coupled utilization of MASSTRIX application [18] for annotation with KEGG Mapper Color application allowed to associate putative identities with molecular pathways. Annotation was performed using specific databases for *Saccharomyces cerevisiae* (model yeast) and *Acetobacter pasteurianus* (acetic acid bacterium). Distribution of putative identities according to metabolic pathways is available in Figure S2. Main metabolic pathways were specific to plants and involved the synthesis of polyphenols and phytochemical. Other common pathways were also represented, such as sugar metabolism or the biosynthesis of cofactors. Pathways exclusive to yeast involved starch and sucrose metabolism, and alpha linoleic acid metabolism, whereas only aromatic compounds degradation pathways were specific to the acetic acid bacterium.

### 2.3. Impact of Production Phases on the Molecular Composition of Kombucha

Firstly, the impact of production phases P1 and P2 was investigated without taking account of the tea type. Therefore, in this part the ST (sugared tea) group corresponded to common formulae of SBT and SGT. The D7 group corresponded to common formulae of BTKD7 and GTKD7 and the D12 group corresponded to common formulae of BTKD12 and GTKD12.

**Figure 3:**
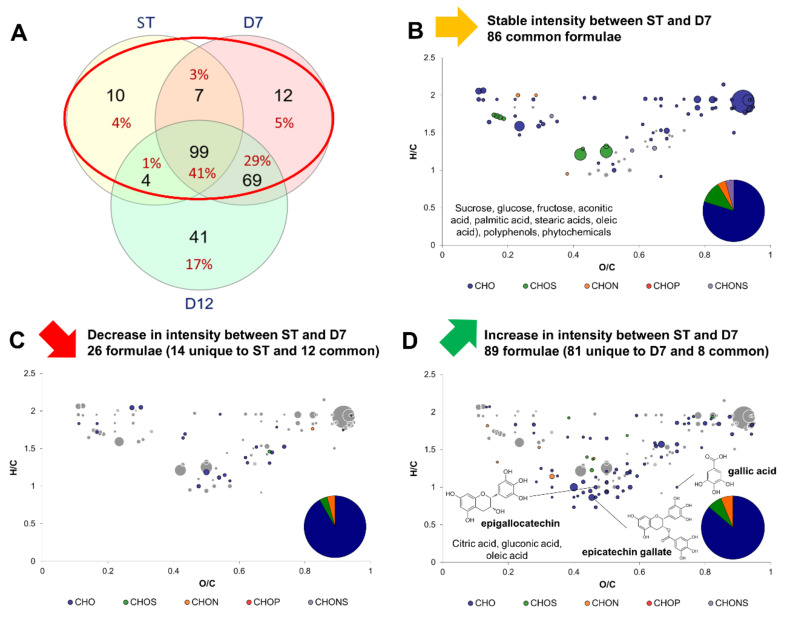
Change in composition between sugared teas (common formula between sugared black and green teas) and day 7 (D7) kombuchas (common formula between black and green tea kombuchas). (**A**) Venn diagram of formulae identified at each process step: Sugared tea (ST), day 7 (D7) and day 12 (D12) with focus on the comparison of ST with D7 (red circle). Van Krevelen diagrams and putative identities of (**B**) stable, (**C**) decreasing and (**D**) increasing formula between ST and D7. The surface of bubbles expresses relative formulae intensity. Core metabolites formulae (Figure 2B) are represented in the background in grey.

Figure 3 shows the modifications induced by P1 by comparing the variations in intensity of formulae between ST and D7. A Venn diagram indicates formulae common or exclusive to ST, D7 and/or D12 (Figure 3A). The core metabolites group of 99 formulae is represented at the intersection of the three ensemble circles. It is clearly visible that the main changes in molecular diversity occurred during P1 with the detection of 81 new formulae in D7 compared to ST. In contrast, only 41 new formulae were detected in D12 exclusively, while 69 were common with D7.

Between ST and D7, formulae that did or did not undergo variations were mainly CHO, and a minority were CHON, CHOS or CHONS, similarly to the core metabolites group (Figure 2B). A total of 86 formulae, including CHONS, remained stable in intensity (no significant difference) and expectedly belonged to the core metabolites group: sucrose, monosaccharides, fatty acids and aconitic acid, the intermediate between citrate and isocitrate in the TCA cycle (Figure 3B). Only 26 formulae decreased in intensity, belonging to small acids, polyphenols, and carbohydrates (Figure 3C). No meaningful putative identity could be included. By contrast, 89 formulae increased in intensity, which belonged mainly to the polyphenol family, namely epigallocatechin (Figure 3D). Other compounds belonged to the carbohydrate family, lipids family (namely oleic acid) but also organic acids: citric, gluconic and gallic acids. Citric acid is namely involved in the TCA cycle, whereas gluconic acid is a biomarker for the oxidative metabolism of acetic acid bacteria and highlights their activity during P1, the acidification phase of kombucha production [9,19]. Finally, increase of gallic acid during kombucha production has been reported in other studies [20,21,22]. Along with the increase in epigallocatechin, this suggests the release of gallic acid through hydrolysis of gallate groups attached to polyphenols via ester bonds (for example epigallocatechin gallate). More evidence regarding this phenomenon were investigated in Section 3.4.

**Figure 4:**
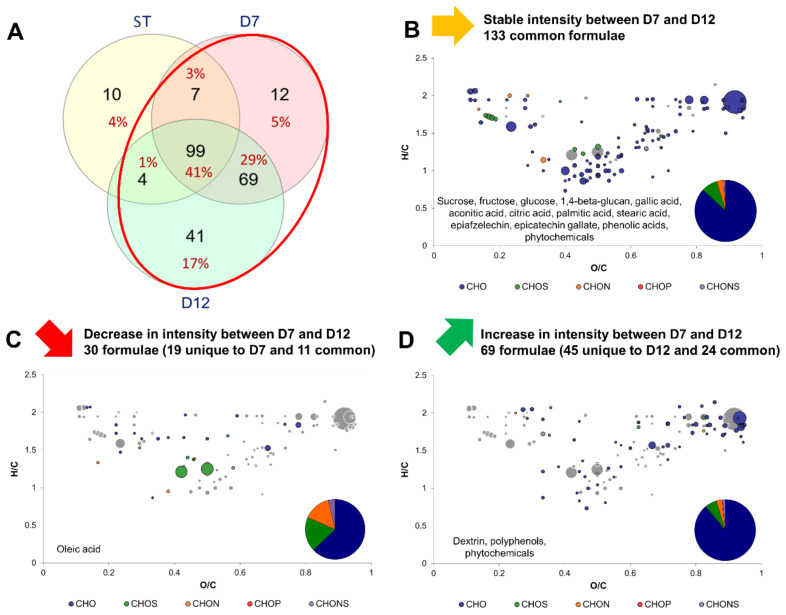
Change in composition between day 7 (D7) and day 12 (D12) kombuchas (common formula between black and green tea kombuchas). (**A**) Venn diagram of formulae identified at each process step: Sugared tea (ST), day 7 (D7) and day 12 (D12) with focus on the comparison of D7 with D12 (red circle). Van Krevelen diagrams and putative identities of (**B**) stable, (**C**) decreasing and (**D**) increasing formula between D7 and D12. The surface of bubbles expresses relative formulae intensity. Core metabolites formulae (Figure 2B) are represented in the background in grey.

Figure 4 shows the modifications induced by P2 by comparing the variations in intensity of formulae between D7 and D12. As stated previously, less new formulae were detected between D7 and D12 compared to between ST and D7 (Figure 4A). Both stable and varying formulae were mainly CHO. However, it is remarkable to note that the proportion in CHON, CHOS and CHONS of decreasing formulae was larger than for stable and increasing formulae during P2. Stable formulae counted 133 and included those of the core metabolites group, but also formulae that increased during P1: citric acid and gallic acid, as well as gallated and non gallated flavan-3-ols (epiafzelechin and epicatechin gallate). The 1,4-beta-glucan can be interpreted as being cellulose since they share the same molecular formula (Figure 4B). Cellulose is a biomarker of acetic acid bacteria metabolism, which produces it from monosaccharides [19]. Decreasing formulae were only 30 and included fatty acids, namely oleic acid, which is an unsaturated fatty acid: constituents of yeasts and acetic acid bacteria plasmic membranes [23,24] (Figure 4C). Therefore, it can be hypothesized that uptake of fatty acids from the medium was stimulated during P2, since oxygen is necessary for the synthesis of unsaturated fatty acids and sterols [25]. It is worth noting that during P1, where microorganism have access to oxygen, oleic acid was produced (Figure 3). Additionally, the production of oleic acid during P1 could be stimulated by the acidification occurring through the oxidative metabolism of acetic acid bacteria. In *Saccharomyces cerevisiae*, oleic acid production was reported to act as a resistance mechanism against acetic acid [26]. Increasing formulae intensities were associated with polyphenols, phytochemicals but also carbohydrates, potentially polysaccharides (Figure 4D).

To sum up, during kombucha production, sucrose, glucose, fructose, and fatty acids such as palmitic and stearic acids remained stable in intensity throughout the two phases. In contrast, polyphenols were widely impacted during the whole process and this suggests a strong impact of microbial activity on their structures as reported in a recent study using UPLC-QTOF-MS [13]. P1 was characterized by the production of gluconic acid through the oxidative metabolism of acetic acid bacteria, but also by the release of gallic acid. The production of those markers did not carry on during P2 and their intensity remained stable. Instead, signs of fatty acids consumption or transformation were observed, probably due to the limitation in oxygen and diverse carbohydrates which appeared to be released.

### 2.4. Impact of Tea Type on the Molecular Composition of Kombucha

**Figure 5:**
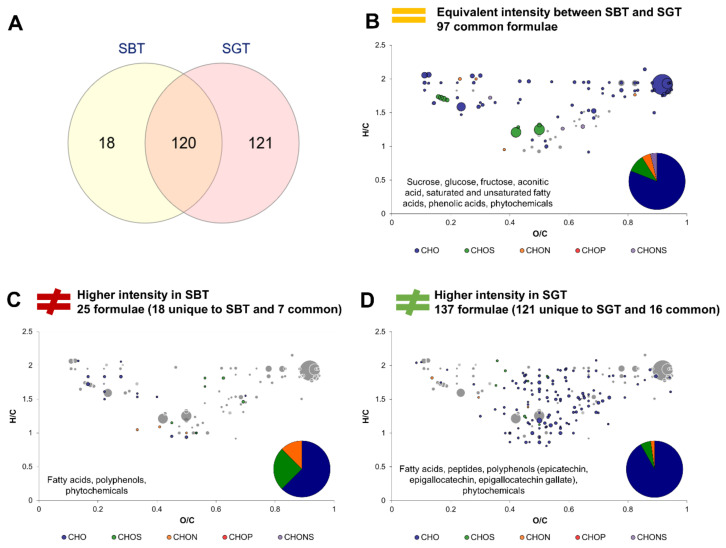
Comparison of composition between Sugared Black Tea (SBT) and Sugared Green Tea (SGT). (**A**) Venn diagram of formulae identified in SBT and SGT. Van Krevelen diagrams and putative identities of formula (**B**) equivalent in intensity in both conditions, (**C**) higher in SBT and (**D**) higher in SGT. The surface of bubbles expresses relative formulae intensity. Core metabolites formulae (Figure 2B) are represented in the background in grey.

Echoing the approach used in Section 3.2., this section investigates the effect of tea type on the molecular composition of kombucha. Firstly, sugared teas SBT and SGT were compared to assess the intrinsic differences of both studied teas (Figure 5). The Venn diagram (Figure 5A) highlights the striking differences regarding unique formulae, with only 18 formulae unique to SBT and 121 to SGT, while 120 were common. Therefore, the green tea used in this study was much richer in terms of molecular diversity than the black tea. Formulae equivalent in intensity belonged to the core metabolites group (Figure 5B). Formulae associated with higher intensities in SBT are CHO in majority, but proportions in CHOS and CHON represented more than 40%. They included fatty acids, polyphenols and phytochemicals (Figure 5C). Formulae with higher intensities in SGT were mainly CHO, with lower proportion in CHOS and CHON. Similar to SBT, SGT distinguished itself through its composition in fatty acids and polyphenols that included epicatechin, epigallocatechin and epigallocatechin gallate, as well as phytochemicals and peptides (Figure 5D).

**Figure 6:**
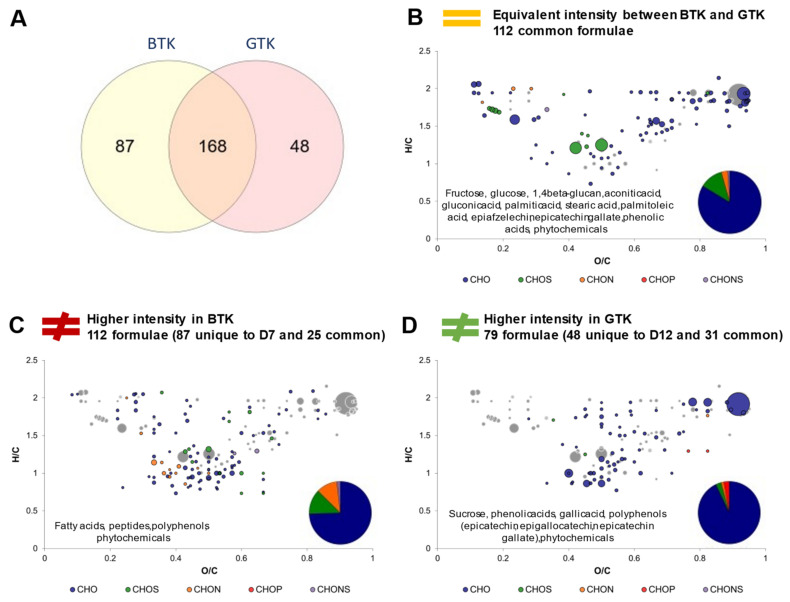
Comparison of composition between black tea kombucha (BTK) and green tea kombucha (GTK) (common formulae between day 7 and day 12). (**A**) Venn diagram of formulae identified in BTK and GTK. Van Krevelen diagrams and putative identities of formula (**B**) equivalent in intensity in both conditions, (**C**) higher in BTK and (**D**) higher in GTK. The surface of bubbles expresses relative formulae intensity. Core metabolites formulae (Figure 2B) are represented in the background in grey.

With the differences between sugared teas in mind, corresponding kombuchas can be compared (Figure 6). In this part, BTK and GTK regrouped for each tea type the common formulae of D7 and D12, so that the impact of the process steps was left aside. For example, BTK corresponded to the common formulae of BTKD7 and BTKD12. The Venn diagram display in Figure 6A strongly contrasts with the one comparing the sugared teas (Figure 5A). While SGT showed higher molecular diversity than SBT, GTK showed less molecular diversity than BTK with, respectively, 48 and 87 unique formulae. Stable formulae in intensity were mainly CHO with small proportions in CHOS, CHON and CHONS (Figure 6B). They include core metabolites formulae as well as carbohydrates such as 1,4-beta-glucan (potentially cellulose), gluconic acid (a marker of acetic acid bacteria activity), palmitoleic acid, phenolic acids, phytochemicals and polyphenols (including flavan-3-ols epiafzelechin and epicatechin gallate). BTK distinguished itself by higher proportions in CHOS and CHON formulae, with specific fatty acids, peptides, polyphenols, and phytochemicals (Figure 6C), while GTK did with phenolic acids, phytochemicals, and polyphenols (Figure 6D). Interestingly, the flavan-3-ols specific to GTK were the same as for SGT (epicatechin, epigallocatechin and epicatechin gallate). In addition, sucrose’s signal was more intense in GTK and CHOP formulae were detected.

To sum up on the effect of tea type on the molecular diversity of kombucha, it appeared that BTK achieved higher molecular diversity than GTK despite SBT having initially lower diversity than SGT. While having common formulae in equivalent amounts, such as fatty acids and flavan-3-ols, BTK and GTK differed significantly on their fatty acids, polyphenols and phytochemical compositions. This highlights the fact that tea types not only affected microbial metabolism, but mainly the interaction between microbial activity and tea compounds, with the generation of different polyphenols and phytochemical derivates. The same phenomenon was also observed in a recent study that compared the polyphenol composition of black and green teas and corresponding kombuchas [13]. Despite the lack of precise microbiological analysis, it can be assumed that another kombucha culture with a different microbial composition from our kombucha culture was used, since they were sourced differently. Therefore, it is worth noting that despite the use of different kombucha cultures, our study also reported higher molecular diversity in polyphenols in black tea kombucha compared to green tea kombucha.

### 2.5. Further Investigations on the Release of Gallic Acid during P1

Release of gallic acid was highlighted during P1 along with potential presence of gallated flavan-3-ol, particularly in SGT and GTK throughout the process. Further investigation regarding the origin of this gallic acid was carried out. The hypothesis was that gallic acid was released from a degallation reaction, meaning the hydrolysis of an ester bound between a gallate group and another molecule, for example a flavan-3-ol such as epicatechin gallate. To achieve this, a Matlab script was conceived to screen formulae with a difference in elemental composition of 7C, 4H and 4O, corresponding to the elemental composition of gallic acid C_7_H_6_O_5_ minus H_2_O consumed by hydrolysis reaction. This returned 63 candidates that were either precursors or products of the degallation reaction. After verification of molecular structures involving ester-bound gallate groups, only three couples could possibly be part of such reaction: epicatechin/epicatechin gallate, epigallocatechin/epigallocatechin gallate and epiafzelechin/epiafzelechin gallate. Positions of gallic acid, epigallocatechin and epicatechin gallate on the Van Krevelen diagram are indicated in Figure 3D. Although none of those putative identities were detected in SBT, all of them were detected in BTK except epicatechin gallate and epiafzelechin gallate (Figure 3). Epigallocatechin and epiafzelechin gallate putative identities were not detected in SGT but all putative identities were detected in GTK with significantly higher intensity if detected in SGT. This means that, when detected, all putative formulae, with or without gallate group, underwent an increase during P1 along with gallic acid. Since monogallated forms did not undergo a decrease or stagnation in intensity, it implies that degallation might occur in digallated forms, or polymeric polyphenols with multiple gallate groups that could not be annotated. Such compounds’ masses might be higher than the range of analysis, or else they did not belong in the databases. It has been hypothesized that polyphenol transformation was induced by microbial activity, either spontaneously due to physical chemical changes, such as the decrease in pH value, or biologically through microbial enzymatic activity, that has yet to be determined [21,27]. Indeed, the pH value of D7 samples were 4.2 ± 0.1, which is higher than the values (between 3.2 and 3.5) reported in other studies that described such phenomenon [13,21]. However, different production parameters explain such differences and all values are included in the range of commercial products (pH values above 3) [10]. Echoing this observations, a two-way interaction between wine polyphenol and gut microbiota has been identified, leading to a regulation of the microbiota and an alteration of polyphenols structures through microbial activities [28]. Although this non-targeted investigation could not conclude on the origin of the gallic acid release during P1, it provided a strong basis for experimentation based on targeted methods. Indeed, the increase of gallic acid content in kombucha is valuable because the lowering of polyphenols’ molecular mass increases their bioavailability as antioxidants in the context of human nutrition. Namely, gallic acid and epicatechin showed better absorption than epicatechin gallate [29]. Thus, further validation investigation is needed to determine the benefits of kombucha microbial activity for the bioavailability of tea polyphenols.

## 3. Materials and Methods

### 3.1. Generation of Biological Samples

Samples analyzed in this study include sugared tea infusion before and after kombucha fermentation. Briefly, sugared black and green tea infusion were produced by steeping 1 g L^−1^ of black tea (Pu’er Grade 1 TN4107) or green tea (Sencha Zhejiang TV4217) from Les Jardins de Gaia© (Wittisheim, France) in boiling water for one hour. After cooling down at room temperature, 50 g L^−1^ blond cane sugar from Ethiquable© (Fleurance, France) was dissolved. Then 12% (*v*/*v*) of 7-day kombucha was added. The mother culture used to produce this 7-day kombucha was obtained from Biomère (Paris, France). Black tea kombucha (BTK) was produced from sugared black tea (SBT) and green tea kombucha (GTK) from sugared green tea (SGT). After inoculation, incubation occurred at 26 °C in static conditions during 7 days for the first phase of production in an open vessel (P1) that corresponded to a biological acidification [10]. Aluminum foil was loosely applied on the bottle neck to prevent contact with particles and insects. Samples obtained at this stage were labelled D7. Then, bottles were tightly capped for 5 days to initiate the second phase of production (P2), corresponding to a natural carbonation. Samples obtained at the end of the process were labeled D12. Each culture was performed in triplicate in 123 mL Boston flasks with a Specific Interfacial Surface (SIS) of 0.01 cm^−1^ [30].

### 3.2. Analytical Methods of Chemical Parameters

Sugar content was measured using Sucrose Glucose Fructose enzymatic kit from Biosentec (Portet-sur-Garonne, France). The pH values were measured with a Mettler Toledo Five Easy pH meter coupled with a LE498 probe and total acidity was determined by titration with 0.1 N NaOH (purity ≥ 98%) and 0.2% phenolphthalein as color indicator, with reagents purchased from Merck (Darmstadt, Germany).

### 3.3. Sample Preparation for Metabolomic Analysis

All reagents were purchased from Fischer Scientifics (Hampton, VA, USA). Formic acid (CAS 64-18-6) mother solution (50% (*v*/*v*) possessed the puriss p.a. grade, and pure methanol (CAS 67-56-1) was UHPLC-MS grade with purity ≥ 99.9%. All samples were centrifuged at 3000× *g* for 10 min at 4 °C to remove cells and particles before freezing at −18 °C. Once all samples were available, solid phase extraction (SPE) was performed using Bond Elut C18 cartridges from Agilent (Santa Clara, CA, USA). The aim of this step was to reduce the amount of sugar in the sample. Sugars are highly ionizable compounds that can suppress the signal of other ion when present in large quantity [31]. Samples were acidified to reach a pH value between 1.5 and 2.0 using 50% (*v*/*v*) formic acid. Then, the column was conditioned using 2 mL of methanol and 1 mL of 2% (*v*/*v*) formic acid. One mL of sample was added, followed by 1 mL of formic acid for washing. Extract was harvested by adding 1 mL of methanol. This extract was then diluted in methanol at the rate of 1/40 (*v*/*v*). Diluted samples were kept at −18 °C before analysis. 

### 3.4. Fourier Transform-Ion Cyclotron Resonance-Mass Spectrometry (FT-ICR-MS)

Ultrahigh-resolution FT-ICR-MS was performed with a 12 T Bruker Solarix mass spectrometer (Bruker Daltonics, Bremen, Germany) equipped with an APOLLO II electrospray source in negative ionization mode [16]. The diluted samples were infused into the electrospray ion source at a flow rate of 120 μL h^−1^. Settings for the ion source were the following: drying gas temperature 180 °C, drying gas flow 4.0 L min^−1^, capillary voltage 3600 V. Spectra were externally calibrated by ion clusters of arginine (10 mg L^−1^ in methanol). Internal calibration of each spectrum was carried out using a reference list including selected markers and ubiquitous fatty acids at 0.1 ppm. The spectra were acquired with a time-domain of 4 megawords and 400 scans were accumulated within a mass range of *m*/*z* 92 to 1000. A routine resolving power of 400,000 at *m*/*z* 300 was achieved [17,32].

### 3.5. Processing of FT-ICR-MS Data 

Raw spectra were post-processed using the software Compass DataAnalysis 4.2 (Bruker Daltonics, Bremen, Germany). Peaks processing was very conservative with a signal-to-noise ratio (S/N) of at least 4 that were exported to mass lists [17]. For all samples, exported *m*/*z* features were aligned into a matrix containing averaged *m*/*z* values (peak alignment window width: ±1 ppm) and corresponding peak intensities. Molecular formulae were assigned to the exact *m*/*z* values by mass difference network analysis using an in-house developed software tool NetCalc [33]. In total, the matrix containing the entire sample set presented 506 detected features that could be assigned to distinct and unique molecular formulae. More than 90% of all assignments were found within an error range lower than 0.2 ppm. All further calculations and filtering were completed in Perseus 1.5.1.6 (Max Planck Institute of Biochemistry, Martinsried, Germany) and R Statistical Language (version 3.1.1). To validate the detection of a mass for a given condition (for example SBT or BTKD7), a given mass had to be detected in at least 2 of the 3 replicates which in consequence left only 471 masses in total. Annotation of formulae was made using the METLIN database and assignation to metabolic pathways was performed using MASSTRIX database coupled with KEGG Color Mapper tool.

### 3.6. Repeatability of FT-ICR-MS Measurements

Quality control (QC) samples were produced by mixing all extract samples in equal amounts. To monitor the reproducibility of the measurements overtime, QC samples were injected at the beginning and after every 10 samples (Figure S1). The coefficient of variation was then calculated from the peak intensities of all elemental compositions detected in the QC samples (Figure S1D). More than 90% of all elemental compositions showed a CV-value lower than 20%. 

### 3.7. Statistical Analysis

Treatment of mass lists and analysis of variance (ANOVA) with α = 0.05 were performed using Perseus 1.5.1.6 (Max Planck Institute of Biochemistry, Martinsried, Germany). Principal Component Analysis (PCA) and Hierarchical Clustering Analysis (HCA) were performed using R software (version 4.0.1). Van Krevelen diagrams (O/C versus H/C elemental ratios) and multidimensional stoichiometric compounds classification (MSCC) have been used to elucidate main compound categories commonly defined as lipids, peptides, amino sugars, carbohydrates, nucleotides and polyphenols compounds [15,31]. Venn diagrams were generated using Molbiotools’ Multiple List Comparator (https://www.molbiotools.com/listcompare.php, accessed on 2 August 2021). Investigation on the release of gallic acid was realized using Matlab (R2015a).

## 4. Conclusions

This study gave extensive insight into the nature of the transformations happening to sugared tea during kombucha production, and how the type of tea influenced them. The biological acidification phase P1 in open vessel was the production step that transformed the matrix most with the production of news compounds including gluconic acid, a marker of acetic acid bacteria activity. This phase was also characterized by the transformation of polyphenols and the release of gallic acid from bound forms. The natural carbonation phase P2 in closed vessel was characterized by a less changes and the consumption of fatty acids in a context of oxygen deprivation. Black tea and green tea induced the production of different metabolites, but black tea kombucha underwent a much stronger modification of polyphenols than green tea kombucha. Those new elements are useful to further define kombucha and guide the conception of its regulation worldwide. New hypotheses were raised and can be used as the basis of further investigation using targeted methods. For example, the mechanisms of polyphenol transformation and its role in potential health benefits of kombucha consumption are a potential topic to cover. However, a similar non-targeted approach could be used to investigate microbial interaction in kombucha with experimental designs involving controlled microbial compositions.

## Data Availability

The data presented in this study are available within the article and supplementary data.

## Supplementary Materials

The following supporting information can be downloaded at: https://www.mdpi.com/article/10.3390/metabo12020160/s1, Figure S1: Visualization of Quality control (QC) samples. (A) maximum, (B) average and (C) minimal ion intensities measured in QC samples. (D) Distribution of mass number according to variation coefficient of QC samples. (E) Principal Component Analysis d1 coordinate of samples according to passage number, with QC samples signalized in red, Table S1: Database annotation of markers, Figure S2: Distribution of annotated compounds using MASSTRIX database according to metabolic pathways according to KEGG Mapper Color.
