# Supplementary material for: Non-Targeted Metabolomic Analysis of the Kombucha Production Process
Source: Metabolites. 2022 Feb 8;12(2):160. doi: 10.3390/metabo12020160 (PMC8878552; doi:10.3390/metabo12020160)
Supplement: Supplementary file 1 [file metabolites-12-00160-s001.zip › metabolites-1575363-supplementary.pdf]

Supplementary Material

Non-Targeted Metabolomic Analysis of the Kombucha Production Process

Thierry Tran [1,*], Rémy Romanet [1], Chloé Roullier-Gall [1], François Verdier [2], Antoine Martin [2], Philippe Schmitt-Kopplin [3,4], Hervé Alexandre [1], Cosette Grandvalet [1] and Raphaëlle Tourdot-Maréchal [1]

[1] UMR Procédés Alimentaires et Microbiologiques, Université de Bourgogne Franche-Comté/Institut Agro Dijon, 21000 Dijon, France; remy.romanet@u-bourgogne.fr (R.R.); chloe.Roullier-Gall@u-bourgogne.fr (C.R.-G.); rvalex@u-bourgogne.fr (H.A.); cosette.grandvalet@u-bourgogne.fr (C.G.); tourdot@u-bourgogne.fr (R.T.-M.)

[2] Biomère, 14 rue Audubon, 75120 Paris, France; fverdier@jubiles.bio (F.V.); amartin@jubiles.bio (A.M.)

[3] Comprehensive Foodomics Platform, Technische Universität München, 85354 Freising, Germany; schmitt-kopplin@helmholtz-muenchen.de

[4] Research Unit Analytical BioGeoChemistry, Department of Environmental Sciences, Helmholtz Zentrum München, 85764 Neuherberg, Germany

* Correspondence: thierrytran01@gmail.com

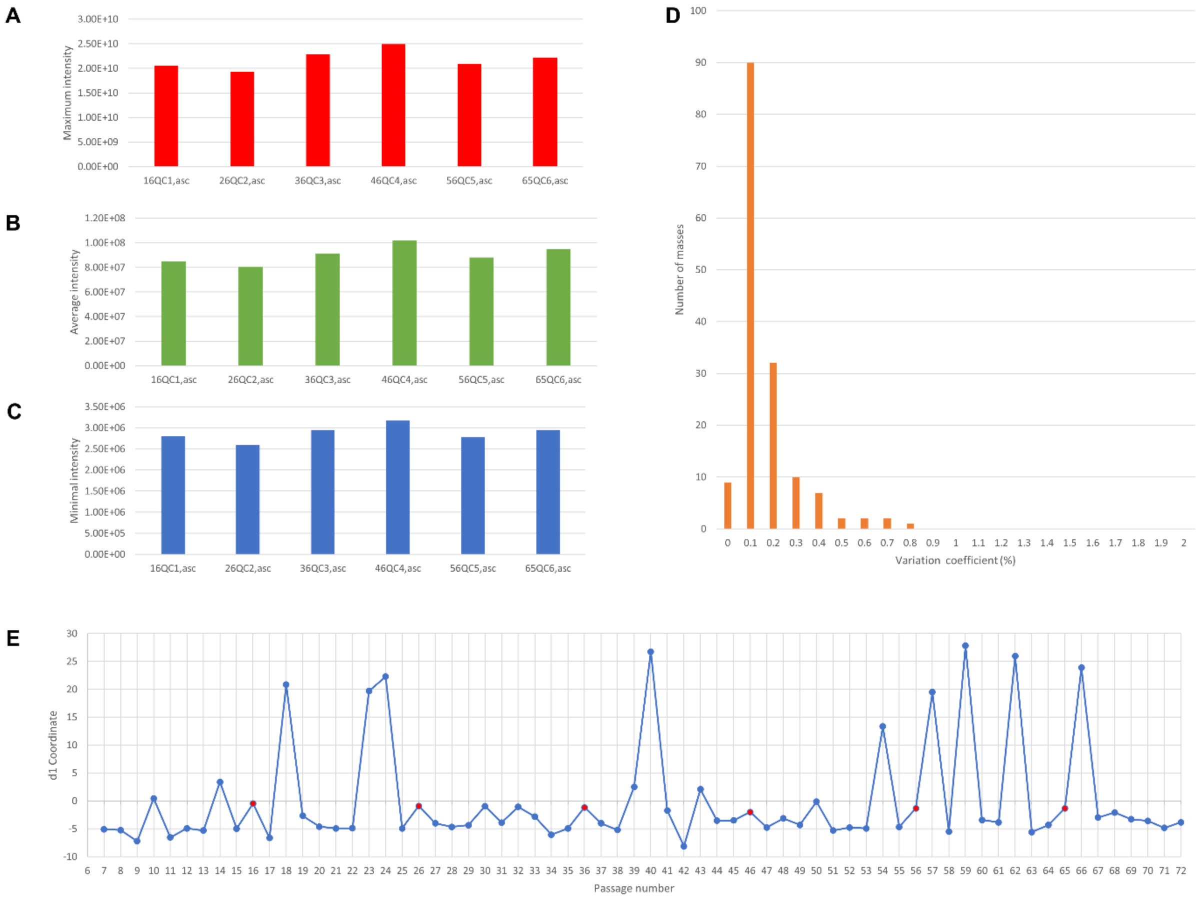

Figure S1: Visualization of Quality control (QC) samples. (A) maximum, (B) average and (C) minimal ion intensities measured in QC samples. (D) Distribution of mass number according to variation coefficient of QC samples. (E) Principal Component Analysis d1 coordinate of samples according to passage number, with QC samples signalized in red.

Table S1: Database annotation of markers

Mass (average)	Formula	Database annotation
169.01427	$C_7H_6O_5$	Gallic acid
173.00917	$C_6H_6O_6$	aconitic acid
179.05612	$C_6H_{12}O_6$	Glucose or Fructose
191.01973	$C_6H_8O_7$	Citric acid
195.05103	$C_6H_{12}O_7$	Gluconic acid
253.21727	$C_{16}H_{30}O_2$	Palmitoleic acid
255.23292	$C_{16}H_{32}O_2$	Palmitic acid
273.07682	$C_{15}H_{14}O_5$	Epiafzelechin
281.24859	$C_{18}H_{34}O_2$	Oleic acid
283.26424	$C_{18}H_{36}O_2$	Stearic acid
289.07175	$C_{15}H_{14}O_6$	Epicatechin
305.06669	$C_{15}H_{14}O_7$	Epigallocatechin
341.10891	$C_{12}H_{22}O_{11}$	Sucrose
425.08788	$C_{22}H_{18}O_9$	Epiafzelechin gallate
441.08278	$C_{22}H_{18}O_{10}$	Epicatechin gallate
503.16189	$C_{18}H_{32}O_{16}$	Dextrin
535.15180	$C_{18}H_{32}O_{18}$	1,4-bêta-D-Glucan

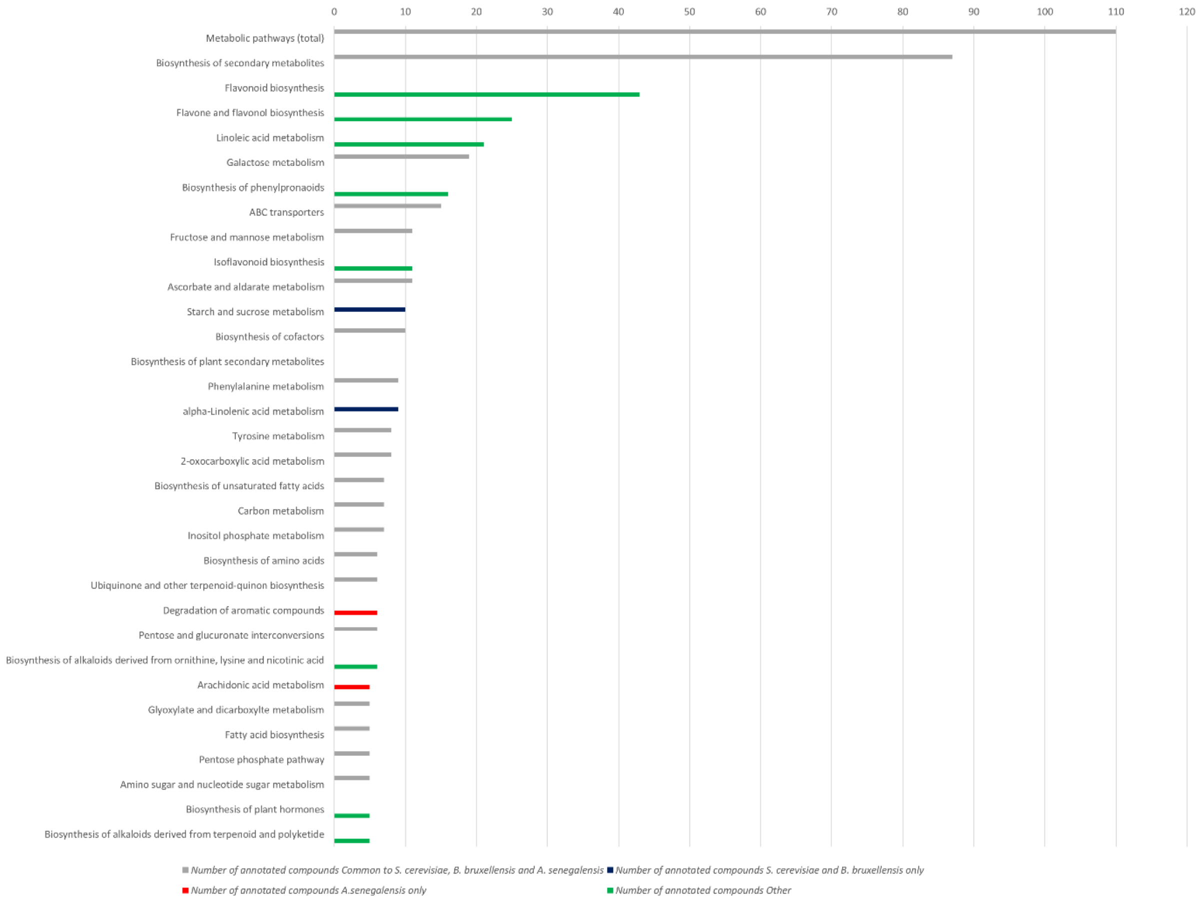

Figure S2: Distribution of annotated compounds using MASSTRIX database according to metabolic pathways according to KEGG Mapper Color.
